# Ethical considerations in malaria research proposal review: empirical evidence from 114 proposals submitted to an Ethics Committee in Thailand

**DOI:** 10.1186/s12936-015-0854-5

**Published:** 2015-09-14

**Authors:** Pornpimon Adams, Sukanya Prakobtham, Chanthima Limphattharacharoen, Pitchapa Vutikes, Srisin Khusmith, Krisana Pengsaa, Polrat Wilairatana, Jaranit Kaewkungwal

**Affiliations:** Office of Research Services, Faculty of Tropical Medicine, Mahidol University, Bangkok, Thailand; Department of Microbiology and Immunology, Faculty of Tropical Medicine, Mahidol University, Bangkok, Thailand; Department of Tropical Pediatrics, Faculty of Tropical Medicine, Mahidol University, Bangkok, Thailand; Department of Clinical Tropical Medicine, Faculty of Tropical Medicine, Mahidol University, Bangkok, Thailand; Department of Tropical Hygiene, Faculty of Tropical Medicine, Mahidol University, Bangkok, Thailand

**Keywords:** Ethics, Developing countries, Malaria, Institutional review board, Proposals

## Abstract

**Background:**

Malaria research is typically conducted in developing countries in areas of endemic disease. This raises specific ethical issues, including those related to local cultural concepts of health and disease, the educational background of study subjects, and principles of justice at the community and country level. Research Ethics Committees (RECs) are responsible for regulating the ethical conduct of research, but questions have been raised whether RECs facilitate or impede research, and about the quality of REC review itself. This study examines the review process for malaria research proposals submitted to the Ethics Committee of the Faculty of Tropical Medicine at Mahidol University, Thailand.

**Methods:**

Proposals for all studies submitted for review from January 2010 to December 2014 were included. Individual REC members’ reviewing forms were evaluated. Ethical issues (e.g., scientific merit, risk–benefit, sample size, or informed-consent) raised in the forms were counted and analysed according to characteristics, including study classification/design, use of specimens, study site, and study population.

**Results:**

All 114 proposals submitted during the study period were analysed, comprising biomedical studies (17 %), drug trials (13 %), laboratory studies (24 %) and epidemiological studies (46 %). They included multi-site (13 %) and international studies (4 %), and those involving minority populations (28 %), children (17 %) and pregnant women (7 %). Drug trials had the highest proportion of questions raised for most ethical issues, while issues concerning privacy and confidentiality tended to be highest for laboratory and epidemiology studies. Clarifications on ethical issues were requested by the ethics committee more for proposals involving new specimen collection. Studies involving stored data and specimens tended to attract more issues around privacy and confidentiality. Proposals involving minority populations were more likely to raise issues than those that did not. Those involving vulnerable populations were more likely to attract concerns related to study rationale and design.

**Conclusions:**

This study stratified ethical issues raised in a broad spectrum of research proposals. The Faculty of Tropical Medicine at Mahidol University is a significant contributor to global malaria research output. The findings shed light on the ethical review process that may be useful for stakeholders, including researchers, RECs and sponsors, conducting malaria research in other endemic settings.

## Background

Malaria research is predominantly conducted in developing countries, corresponding to endemic areas of the disease. Over the past few decades, there have been ongoing concerns and arguments on research ethics. These issues are related to the over- or under-protection of human subjects in drug and vaccine clinical trials, as well as non-clinical studies carried out in disease burden areas [1–6]. Concerns raised include the balance between ethics and science, patient risks and benefits, individuals’ educational background and local concepts of health and disease, ethical justice principles at the community and country level, and even arguments around the Hippocratic dictum ‘*primum non nocere’* [1, 2, 5]. Regarding research ethics in developing countries, it is a reasonable notion that regardless of where the research is conducted, not only should the quality be the same, but also that study participants are equally valued and respected [7]. Despite the generic ethical principles that can be applied to healthcare-related research, there has been debate about the application of such principles in different research settings because socio-cultural and economic contexts vary considerably around the world [2, 7].

In attempting to prohibit malpractice of human experiments in the medical community, the “Declaration of Helsinki” was initially adopted in 1964 and has undergone several revisions since. The Declaration introduced the concept of an independent committee; it stated explicitly that the research protocol must be submitted to the concerned research ethics committee for consideration, comment, guidance, and approval before the study begins [8]. The research ethics committee (REC) must be qualified, independent, and have no conflict of interest when reviewing the research protocol. This evolved into the term “institutional review board” (IRB) used in the US. The IRB concept was established after the U.S. National Research Act (1974), and the Belmont Report (1979), stating its purpose to protect human subjects involved in both clinical and behavioural research [9]. The IRB Guidebook [10] was developed by the US Office for Human Research Protections (OHRP) and is one of the resources that ethics committees across the globe adopt for their own operations. It includes issues of IRB administration, regulation and policies, protocol review mechanisms and other ethical principles. Similarly, the European Union Clinical Trials Directive regards the research ethics committee as an independent body with responsibility to protect the rights, safety, and well-being of human subjects involved in a research study [11]. The terms IRB and REC are often used interchangeably, but arguably have somewhat different meanings; according to the ICH Guideline [12]: IRB could be a subsection of REC (a review board or a committee, institutional, regional, national, or supranational), but the IRB or REC plays a major role in regulating the ethical conduct of research by reviewing research proposals before the research is carried out. While judging whether the proposal is worthwhile and ethical, part of the committee’s role may also be to provide constructive recommendations to researchers in an attempt to maintain high-quality, ethical research [8, 10–12].

With its role in ethical review of research proposals, questions have been raised about whether the REC is facilitating or impeding the advancement of scientific research, and there have been comments from researchers regarding the quality of the REC review itself [13]. Some investigators in international or multicentre studies have complained of substantial inefficiencies in REC review, and have requested that RECs pay more attention to scientific integrity alongside the protection of human subjects involved in the study [14]. Investigators have also raised concerns that REC review burdens have grown to include studies involving interviews, secondary use of public-use data and similar activities, most of which involve minimal risks [9]. Criticism has also been raised regarding the REC requirement for paperwork and mechanical monitoring, which may undermine the main goal of the protection of human subjects [9]. With the emergence of new fields in biomedical research and technology, particularly in genetics and genomics, a range of views exist within and between RECs and the research groups in these evolving fields [15]. This reflects the complexity and diversity and lack of common ground surrounding many ethical issues related to this type of research [15, 16].

In assessing ethical quality in protocol review—particularly the oversight of human subject research—RECs and other stakeholders, including sponsors, regulators and the public, need to have evidence-based information [17, 18]. Several proxy indicators of ethical quality assessment have been proposed. Some suggest using measurement parameters and/or quality metrics including objective and subjective quality assessment, which can be used to improve REC review and deliberation processes, and strengthen relationships between the REC and researchers [13, 19]. It has been suggested that ethnographic studies on REC meetings and minutes could help identify the critical ethical issues that the REC considers when reviewing proposals, thus determining whether ethical principles have been thoroughly considered [14].

An analysis of malaria publication data (2010–2014) in the SciVal database, to which Mahidol University subscribes, found that Mahidol University (Thailand) ranked #4 (with 450 papers) for number of research studies. Of these, the Faculty of Tropical Medicine, Mahidol University contributed 87 % (with 391 papers), correlating to a ranking of #6 if counted as an independent institute. To promote the conduct of research at FTM, the Office of Research Services (ORS) provides administrative services to the faculty’s research community. One of its major functions is as the Secretariat to the FTM Ethics Committee (FTM-EC), managing the operations of Ethics Committee meetings. The FTM-EC has been continuously registered with the Federal-wide Assurance (FWA) of the US OHRP since 2002. The FTM-EC comprises one clinical and one non-clinical panel, which convene at monthly meetings.

Given the level of malaria research output and ethics committee activity at the FTM, it is an ideal location to examine the REC review process for malaria research. Therefore, this study attempts to reveal how malaria research studies conducted in Southeast Asia (along the Thai borders) were reviewed by the local REC, the FTM-EC. In particular, the purpose of this study was to identify the ethical considerations for different types of malaria research proposals submitted to the FTM-EC during the period 2010–2014.

## Methods

### Dimensions of ethical consideration

In reviewing research proposals, REC members should base their judgment on international standards of ethical concerns pertaining to moral values including dignity, bodily integrity, autonomy, and privacy. Such ethical concerns are listed in several guidelines for REC review, and although they might be expressed differently in varying circumstances, similar principles are often cited [11, 20–24]. The World Health Organization (WHO) [22] states in its standards for ethical review that the approval or disapproval of the protocol should be based on the ethical acceptability of the research while accounting for its social value, scientific validity, and applicable laws. As stated in the guidelines of the Council for International Organizations of Medical Sciences (CIOMS) [21], all research involving human subjects should be in accordance with the moral weight of the three basic ethical principles: respect for persons, beneficence, and justice. The European guidelines [11] suggest four main components of a research ethics committee: dignity, bodily integrity, autonomy, and privacy. The European Commission [23] even suggests that researchers pre-check their proposal to ensure that it follows the principles of human dignity, fair distribution of research benefits and burden, and protection of the values, rights, and interests of the research participants. The UK Health Department [24] provides guidance in governance arrangements for RECs in reviewing research proposals to act as part of an efficient, accountable, and independent body in protecting the dignity, rights, safety, and well-being of people who take part in research. The US IRB Guideline on basic IRB review [25] discusses issues of human subject research, in particular for risk–benefit analysis, monitoring and observation, informed consent and additional safeguards, selection of subjects, incentives for participation, and privacy and confidentiality.

Ethics is not about prescribing a specific set of rules or policies, rather it is about providing a framework for evaluating problems and determining an appropriate course of action [26]. According to the WHO manual for capacity training for RECs [26], the ethical analysis is to identify a set of governing principles reflecting both internationally accepted norms and locally relevant cultural values, and then apply such principles to evaluate the research. Several frameworks for ethical research conducted in developing countries have been proposed, focusing on collaborative partnerships and sharing responsibilities with researchers, policy-makers and the community [2, 21, 26, 27]. The framework should be based on the notation set forth in the Declaration of Helsinki so that ethical principles could be effectively identified and applied, particularly in developing countries in regards to each country’s socioeconomic circumstances, laws and regulations, and executive and administrative arrangements [21]. Proposed basic ethical principles include: (1) considering social value by specifying the beneficiaries of the research, (2) ensuring scientific validity through the scientific design and research objectives, (3) fair study population selection to ensure scientific validity, (4) assessment of the risk–benefit ratio by comparing the net risks of the research project with the potential benefits, (5) ensuring public accountability through independent reviews mandated by laws and regulations, (6) ensuring informed consent while involving the community in establishing recruitment procedures and incentives, and (7) respecting study participants and communities [2, 26, 27].

In particular, ethical issues that should be considered as part of malaria field studies in developing countries have been discussed as lessons learned in a community-based clinical trial of rectal artesunate conducted in a few developing countries [28]. Issues to consider include the ethical review process, standard of care, incentives and reimbursement, and insurance and indemnity. It has also been suggested that local ethical review should consider the vulnerability of patients with no or poor access to healthcare, specific cultural attitudes, literacy, and both local written and oral languages [28]. A study on convergent ethical issues in HIV/AIDS, TB, and malaria vaccine trials in Africa revealed that sharing simple and effective consent form templates and procedures across diseases was achievable [29]. Comprehension testing of subjects prior to and during study participation, to ensure understanding of the important study concepts, has also been proposed [28]. Moreover, it has been suggested that the REC should also examine the suitability of the investigators and the adequacy of facilities and the methods and documentation to be used in the study [11].

At the FTM-EC, individual REC members are provided with a review form prior to every convened meeting. The review form outlines certain ethical issues similar to those described above. Each member uses this form as a guide to ethical issues to consider when reading the proposal. They then complete each part that he/she considers relevant and requires explanations from the researchers. The form also has an open-ended component, where the REC can note other ethical considerations. The review form used during the study period (currently the form has been revised) guiding the REC members in reviewing each protocol was composed of 19 check boxes for close-ended items on being a human research subject study: types of study (clinical, epidemiological, social science, or behavioural); reasonable scientific questions/objectives; proper sampling techniques/data collection methods; quality of investigators/facilities; project budget; compensation; justification of the involvement of vulnerable populations; rationale for use of human specimen; adequate toxicological/pharmaceutical information; sufficient provision of information and proper informed consent process and forms; rating on level of risk and benefit of the study; and the overall ethical acceptability of the proposal. The review form also consists of 9 open-ended comment items regarding project summary (to be filled in by two primary reviewers) and other ethical issues (to be filled by all REC members) including major points of concern, levels of risk, comments on the title, proposal content (objectives, research methodology, protection of privacy and confidentiality), participation information sheet, informed consent/assent form, questionnaire/advertisement/case record form, attached document (investigator’s brochure, material transfer, etc.), and other comments/suggestions. For the purpose of this study, the ethical issues were divided into six dimensions, as follows: (1) study rationale and validity; (2) study design; (3) study participants; (4) informed consent process; (5) data collection and analysis; and (6) facility and support. Each dimension thus covers its related ethical considerations in the review form (as presented in the “Results” section).

### Classification of malaria research studies

Research protocols reviewed by RECs can be categorized according to different schemes [22, 30, 31]. The European Science Foundation [31] reviews health research classification systems in different countries across continents. The UK Health Research Classification System (HRCS) for classifying and analyzing biomedical and health research funding applies a two-dimensional framework. Codes on health categories are used to classify the type of health or disease being studied, covering 21 categories encompassing all diseases, conditions and areas of health. Research activity codes are used to classify the type of research activity being undertaken (from basic to applied), covering 48 codes of eight subgroups: (1) Underpinning, (2) Aetiology, (3) Prevention, (4) Detection and Diagnosis, (5) Treatment Development, (6) Treatment Evaluation, (7) Disease Management, and (8) Health Services. The Australian and New Zealand research classification scheme has been developed and updated over the years. The three constituent classifications in use are: (1) Type of Activity (ToA)—pure basic research, strategic basic research, applied research and experimental development; (2) Field of Research (FoR)—methodology used in research and development (R&D) fields of the research investigated by institutions and organizations as well as emerging areas of study; and (3) Socio-economic objective (SEO) consisting of discrete economic, social, technological or scientific domains for identifying the principal purposes of R&D.

On the other hand, the WHO [22] simply classifies different types of research studies to be reviewed by RECs, including, but not limited to, the following: (1) clinical trials, (2) epidemiological research, (3) social science research, (4) research on medical records or other personal information, (5) research on stored samples, (6) health systems research, and (7) implementation research. The US Office for Human Research Protections [30] categorized research reviewed by IRBs as either biomedical or behavioural studies. Biomedical research covers two types of studies: (1) those designed primarily to generate scientific knowledge about the natural history of the disease and normal or abnormal physiology, and (2) studies designed primarily to evaluate the development of medical products and the efficacy, effectiveness, efficiency and safety of a medical intervention. Behavioural research includes studies of the epidemiology and social science of individual or group behaviour. As part of the biomedical research study, clinical trials, which often originate in the laboratory to develop new therapies or procedures, are tested in animal studies, then subsequently on human subjects [32]. The US National Institutes of Health (NIH) distinguishes between different types of clinical trials including, for example, natural history studies, prevention, screening, diagnostic, treatment, or quality of life trials [32]. Biomedical research can be sub-classified as basic/pre-clinical research or clinical research [33].

Many RECs face challenges in the protocol review of studies in the fields of molecular and genetic or genomic research. Lack of clarity on how researchers should respond to RECs has been reported, particularly concerning issues of informed consent and the use of archived specimens [20, 34]. Confusion and debate remains within and across RECs around studies involving the collection and use of non-identifiable stored tissue specimens. There is disharmony among regulatory requirements in different countries and REC bodies within countries, but there is a growing international agreement on the provision and access to research data and bio-specimen collections in order to optimize their long-term value and potential for health discovery and validation [35–37]. The UK Medical Research Council [35] proposed operational and ethical guidelines for the use of human tissue and biological samples, such that samples of human biological material should be treated as donations, and research involving these should be conducted with respect and transparency. The research study should be planned with respect to the trust of the potential donors with individual, cultural, or religious differences in the meaning and significance attached to samples for use.

With contradictory legal and ethical frameworks across national borders, there has been an attempt to set up an international charter of principles for sharing bio-specimens and data [36]. The following five principles include major ethical considerations: (1) respect for privacy and autonomy, (2) reciprocity, (3) freedom of scientific enquiry, (4) attribution, and (5) respect for intellectual property. In the US, the Food and Drug Administration has set rules and regulations regarding the use of identifiable and unidentifiable specimens for clinical investigators, sponsors, and RECs. The US Health Insurance Portability and Accountability Act privacy rule has set less restrictive rules for the use of stored specimens and tissue repositories (such as biobanks), when released data have been de-identified [20]. The UK Medical Research Council [35] emphasizes custodianship as the responsibility of researchers for safe keeping of samples and control of their use and eventual disposal, defining “anonymized samples or data” where all identifying information is removed, either as linked or unlinked anonymous data and samples; and “coded samples or data” where a code is used in place of identifiers to protect the confidentiality of the individual during routine use. The American Society of Human Genetics distinguishes between retrospective and prospective studies, such that the “retrospective research studies” utilise previously obtained samples collected for a purpose that is different from that of the current proposal and the “prospective research studies” are those in which the collection of the new samples is part of the current study design [37]. Similarly, four types of sample identification were defined: (1) anonymous, no identifiers and impossible to link with their sources, (2) anonymised, originally with identified information but irreversibly stripped off and are impossible to link to their sources, (3) identifiable, unidentified for the current research purposes, but can be linked to their sources, and (4) identified, with identifiers and are attached and available to the researchers [37]. In Thailand, however, the use of specimens or medical records, either identifiable or unidentifiable, is considered to be human subject research. Studies involving such material commonly require REC review, either full-board or expedited, and very few receive exemption.

The two panels of the FTM-EC comprise different sets of members. The “clinical panel” reviews clinical research studies involving the application of any clinical interventions in human research subjects, while the “non-clinical panel” reviews other types of biomedical study, including research conducted in clinical settings but where no clinical intervention was applied, epidemiological studies, and studies that use stored specimen or secondary data. Malaria study proposals submitted to the FTM-EC fall into either of the categories and are reviewed by the relevant panel. Because these two broad categories of research might be subject to different constraint levels of ethical consideration, reviewed studies were classified for the purposes of this study in two ways. For classification based on research study design, there are four categories: (1) clinical (drug) trials; (2) biomedical studies; (3) laboratory studies, and (4) epidemiological/social science studies. For classification based on the use and non-use of specimens, there are four categories: (1) new specimen collection; (2) use of archived un-identifiable/un-linked specimens; (3) use of archived/identifiable/linked specimens; and (4) use of medical records and new data collection.

### Sources of information and statistical analysis

This study adopts the process of internal audits on quality systems of independent ethics committees in Europe [38], by conducting documentation reviews. The reviewed documentation included minutes of meetings and agendas, each individual REC member’s reviewing form, and the notifications to researchers informing them of the review outcome for the submitted proposal. This study was conducted by the office managing the submitted proposals and the REC members at the FTM-EC. Information was extracted by personnel authorized to access these documents. To avoid bias, three office employees (non-voting members of the FTM-EC), were assigned to identify the main ethical considerations noted by each REC member on his/her review form, while cross-checking with each other. Ethical considerations raised were counted quantitatively whether the ethical issues (e.g. scientific merit, risk and benefit, sample size, or informed-consent process) were noted/discussed, both in the pre-specified and open-ended items, on each individual REC member’s review form as well as all other documents related to the submitted proposal. It should be noted that for the open-ended items, very little subjective judgment was required by the person extracting the data, because most of the major ethical issues raised by the reviewers could be obviously categorized. All proposals related to malaria research submitted to FTM-EC over a period of 5 years, from January 2010 to December 2014, were reviewed. Analyses of ethical considerations were presented according to study classification, study design and specimen uses; Chi square tests were performed on each ethical issue separately. In addition, analyses were performed according to study location (multi-site, international, approval together with other REC study) and study population (minority or vulnerable population involved). To further investigate the relationship between ethical considerations and different study types, additional statistical analyses were performed using simple crude odds ratios (ORs). Logistic regression was performed for each ethical issue by different type of study separately.

## Results

### Types of malaria research studies

During the study period, 114 research proposals on different malaria species were submitted to the FTM-EC for review. These comprised 19 (17 %) biomedical studies, 15 (13 %) drug trials, 28 (24 %) laboratory studies and 52 (46 %) epidemiological/social science studies. Drug trials adopted atypical phase classification of the drug development process including 12 studies on investigational new drug ranging from Phase I to Phase III, and 3 Phase IV drug safety studies. No malaria vaccine trial proposals were submitted to the FTM-EC during the study period. Biomedical studies included eight studies comparing different malaria treatments/regimens and/or with different indications in clinical settings, and 11 bioequivalence or pharmacokinetic studies. Laboratory studies covered pure basic science and genetic studies. Epidemiology/social science studies included retrospective study using medical records and social/behavioural research. Approximately one-third of studies required FTM-EC approval together with that of other RECs, within Thailand and/or internationally. Approximately 13 % were multi-site and 4 % were international studies. Most malaria studies were conducted at malaria-endemic areas along the Thai borders; therefore, 28 % involved minority (hill tribe and cross-border) populations. Seventeen percent involved children and 7 % involved pregnant women (Table 1).

**Table 1:** Malaria study proposals submitted to the FTM-EC during the study period

Study characteristic	All studies		Biomedical		Drug trial		Laboratory		Epidemiology	
	n = 114		n = 19		n = 15		n = 28		n = 52	
	n	%	n	%	n	%	n	%	n	%
Types of malaria										
*Plasmodium falciparum*	43	37.7	4	21.1	9	60.0	6	21.4	24	46.2
*Plasmodium vivax*	23	20.2	5	26.3	3	20.0	4	14.3	11	21.2
*P. falciparum/P. vivax*	23	20.2	5	26.3	3	20.0	7	25.0	8	15.4
Other	7	6.1	0	0.0	0	0.0	6	21.4	1	1.9
Unspecified	18	15.8	5	26.3	0	0.0	5	17.9	8	15.4
Study type										
International	5	4.4	1	5.3	1	6.7	0	0.0	3	5.8
Multi-centre/-site study	15	13.2	3	15.8	8	53.3	0	0.0	4	7.7
Required other IRB review	35	30.7	8	42.1	11	73.3	7	25.0	9	17.3
Vulnerable subject involvement										
Minority (border areas)	32	28.1	9	47.4	10	66.7	7	25.0	6	11.5
Pregnant women	8	7.0	1	5.3	0	0.0	0	0.0	7	13.5
Children	19	16.7	4	21.1	2	13.3	7	25.0	6	11.5
Unconscious patients	2	1.8	1	5.3	0	0.0	0	0.0	1	1.9
Elderly	2	1.8	0	0.0	0	0.0	1	3.6	1	1.9

*FTM*-*EC* Faculty of Tropical Medicine Ethics Committee and *IRB* institutional review board

### Overall ethical considerations on malaria proposals

**Fig. 1:**
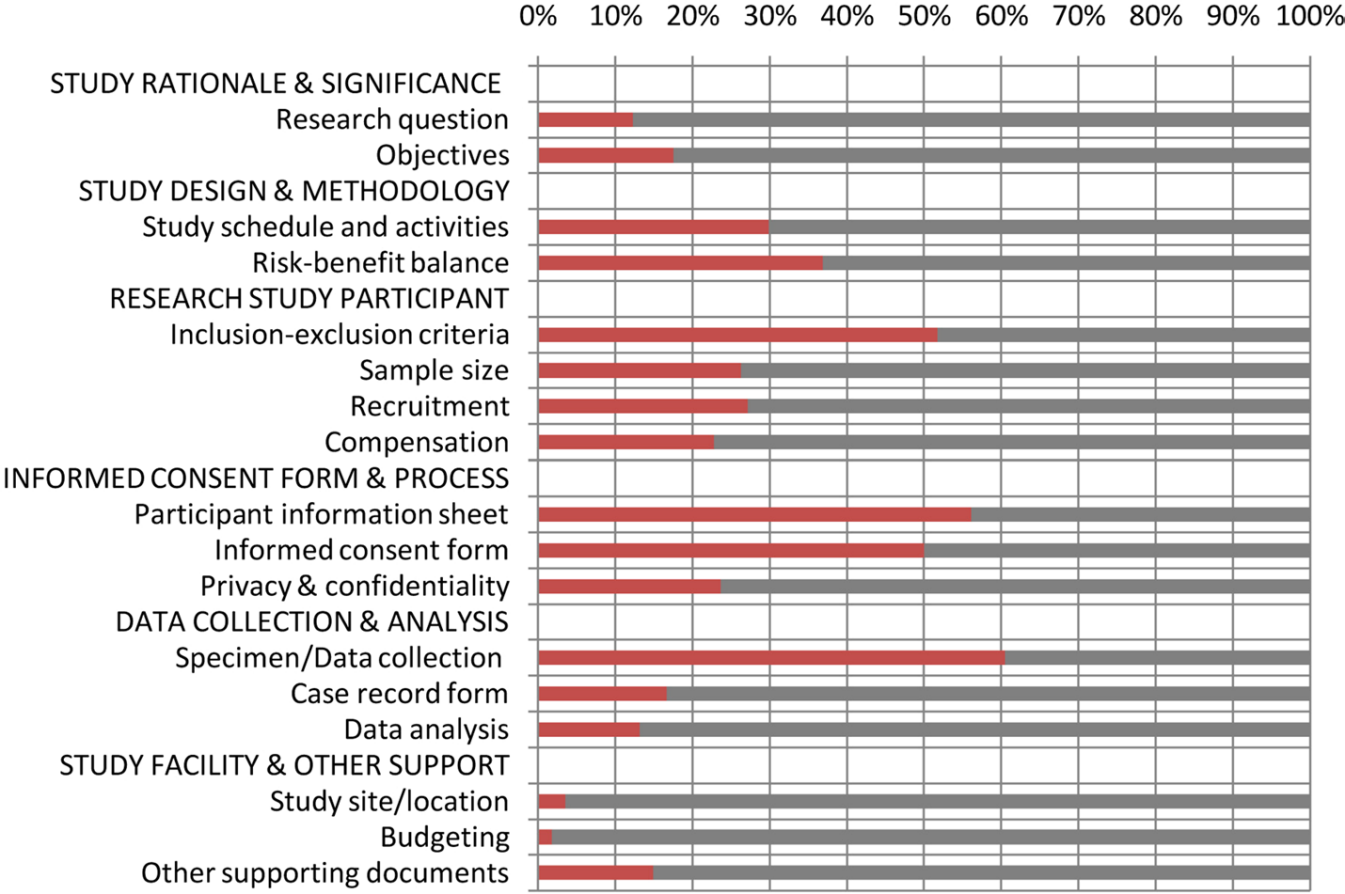
Overall ethical considerations on malaria research proposals (n = 114)

Of the 114 proposals, the ethical issues raised by REC members on “study rationale and significance of the study” included 12 % on research questions and 17 % on research objectives. As shown in Fig. 1, regarding the “study design and methodology” dimension, approximately 30 % were related to study schedule and activities, and 37 % to risk–benefit balance. For the ethical considerations concerning “research study participant”, 52 % were related to inclusion and exclusion criteria, 26 % to sample size, 27 % to recruitment procedures, and 23 % to participant compensation. Higher percentages were shown for the “informed consent process” dimension, with 57 % on the participation information sheet, 50 % on the informed consent form/process, and 24 % on the privacy and confidentiality of the information. Regarding the “data collection and analysis” dimension, approximately 61 % related to the specimen and data collection procedure, 17 % to case record form (CRF) design and use and 13 % to data analysis methods. For “study facility and supporting information”, 4 % were on study site location, 2 % on study budget, and 15 % on supporting documentation (e.g. material transfer agreement, and approval of authorities in the community where the study was taking place).

### Comparisons of ethical considerations by proposal type

When comparing ethical considerations according to study design, almost all ethical issues were statistically significantly different across the four study designs. Proposals for drug trials had higher percentages for most ethical issues, followed by proposals of for biomedical/clinical research then laboratory or basic science research (Table 2). Proposals for epidemiology studies had fewer ethical issues raised by the FTM-EC. Interestingly, ethical issues for “privacy and confidentiality” specifically showed an opposite but non-statistically significant trend, being higher for laboratory and epidemiology studies than biomedical studies or drug trials. Other non-statistically significant differences among different study designs were for CRFs, data analysis, study budget and supporting documents.

**Table 2:** Comparisons of ethical considerations by study design

Ethical issue	Biomedical		Drug trial		Laboratory		Epidemiology		p value
	n = 19		n = 15		n = 28		n = 52		
	n	%	n	%	n	%	n	%	
Rationale and significance									
Research question	6	31.6	2	13.3	1	3.6	5	9.6	0.03
Objectives	5	26.3	6	40.0	6	21.4	3	5.8	0.01
Study design									
Study schedule and activities	7	36.8	9	60.0	10	35.7	8	15.4	0.01
Risk–benefit balance	13	68.4	10	66.7	12	42.9	7	13.5	<0.01
Study participants									
Inclusion/exclusion criteria	11	57.9	11	73.3	20	71.4	17	32.7	<0.01
Sample size	11	57.9	6	40.0	7	25.0	6	11.5	<0.01
Recruitment	5	26.3	5	33.3	13	46.4	8	15.4	0.03
Compensation	6	31.6	10	66.7	7	25.0	3	5.8	<0.01
Informed consent process									
Participation information sheet	17	89.5	14	93.3	21	75.0	12	23.1	<0.01
Informed consent form	13	68.4	14	93.3	21	75.0	9	17.3	<0.01
Privacy and confidentiality	2	10.5	1	6.7	10	35.7	14	26.9	0.08
Data collection and analysis									
Specimen/data collection	15	79.0	14	93.3	17	60.7	23	44.2	<0.01
Case record form	4	21.1	1	6.7	4	14.3	10	19.2	0.64
Data analysis	1	5.3	5	33.3	4	14.3	5	9.6	0.07
Facility and support									
Study site/location	3	15.8	1	6.7	0	0.0	0	0.0	0.01
Budgeting	0	0.0	1	6.7	1	3.6	0	0.0	0.27
Other supporting documents	2	10.5	4	26.7	5	17.9	6	11.5	0.46

When comparing ethical considerations by different types of specimen/data collection and use, again almost all ethical issues were statistically significantly different across the four types of study proposal. Proposals involving new specimen collection had higher percentages for most ethical issues, followed by proposals using medical records or collecting new data using CRFs or questionnaires (Table 3). The proposals involving the use of stored specimens, either linked (identifiable) or unlinked (un-identifiable) had a smaller percentage of ethical issues raised by the FTM-EC. Again, ethical issues for “privacy and confidentiality” showed an opposite but non-statistically significant trend, with the percentage higher for studies involving stored specimens. Other non-statistically significant differences among different study designs were study rationale, data analysis, and study facility and supporting documents.

**Table 3:** Comparisons of ethical considerations by type of specimen/data use

Ethical issues	New specimen		Archived (linked)		Archive (unlinked)		Medical records/CRF		p value
	n = 58		n = 11		n = 20		n = 25		
	n	%	n	%	n	%	n	%	
Rationale									
Research question	9	15.5	1	9.1	3	15.0	1	4.0	0.49
Objectives	14	24.1	1	9.1	1	5.0	4	16.0	0.21
Study design									
Study schedule and activities	27	46.6	0	0.0	3	15.0	4	16.0	<0.01
Risk–benefit balance	37	63.8	0	0.0	2	10.0	3	12.0	<0.01
Study participants									
Inclusion/exclusion criteria	41	70.7	3	27.3	4	20.0	11	44.0	<0.01
Sample size	22	37.9	3	27.3	3	15.0	2	8.0	0.02
Recruitment	25	43.1	2	18.2	0	0.0	4	16.0	<0.01
Compensation	23	39.7	0	0.0	1	5.0	2	8.0	<0.01
Informed consent process									
Participation information sheet	51	87.9	2	18.2	1	5.0	10	40.0	<0.01
Informed consent form	46	79.3	2	18.2	1	5.0	8	32.0	<0.01
Privacy and confidentiality	10	17.2	3	27.3	7	35.0	7	28.0	0.38
Data collection and analysis									
Specimen/data collection	45	77.6	6	54.6	7	35.0	11	44.0	<0.01
Case record form	9	15.5	0	0.0	1	5.0	9	36.0	0.01
Data analysis	9	15.5	2	18.2	0	0.0	4	16.0	0.29
Facility and support									
Study site/location	4	6.9	0	0.0	0	0.0	0	0.0	0.26
Budgeting	2	3.5	0	0.0	0	0.0	0	0.0	0.58
Other supporting documents	12	20.7	1	9.1	0	0.0	4	16.0	0.15

*CRF* case record form

### Strength of relationship between ethical considerations and different types of malaria proposals

Regarding study location, proposals that required other REC review had higher ORs compared with proposals that required only FTM-EC review for the following ethical issues: study design and methodology; study participants; the informed consent process; and study facility and supporting documents. For comparisons between international vs. local studies, and between multi- vs. single-site studies, higher ORs were found for the dimension study facility and supporting documents (Table 4).

**Table 4:** Comparisons of ethical considerations by different study characteristics

Study type	N	Rationale			Study design			Study participants		
		n	%	OR (95 % CI)	n	%	OR (95 % CI)	n	%	OR (95 % CI)
Study locations										
International										
Y	5	3	60.0	4.3 (0.7–27.3)	4	80.0	4.5 (0.5–42.0)	4	80.0	1.9 (0.2–17.6)
N	109	28	25.7	1	51	46.8	1	74	67.9	1
Multi-centre/-site										
Y	15	7	46.7	2.7 (0.9–8.3)	10	66.7	2.4 (0.8–7.5)	13	86.7	3.4 (0.7–15.9)
N	99	24	24.2	1	45	45.5	1	65	65.7	1
Additional review by other IRB										
Y	35	12	34.3	1.7 (0.7–3.9)	22	62.9	*2.4 (1.0–5.3)*	30	85.7	*3.9 (1.4–11.1)*
N	79	19	24.1	1	33	41.8	1	48	60.8	1
Study population										
Involved minority populations										
Y	32	12	37.5	2.0 (0.8–4.8)	26	81.3	*7.9 (2.9–21.5)*	31	96.9	*23.1 (3.0–177.3)*
N	82	19	23.2	1	29	35.4	1	47	57.3	1
Involved vulnerable populations										
Y	28	13	46.4	*3.3 (1.3–8.1)*	19	67.9	*2.9 (1.1–7.2)*	22	78.6	2.0 (0.7–5.4)
N	86	18	20.9	1	36	41.9	1	56	65.1	1
Clinical study design										
Biomedical studies	19	10	52.6	*6.1 (1.9–19.8)*	15	79.0	*11.3 (3.2–40.0)*	16	84.2	*7.3 (1.9–28.1)*
Drug trial	15	6	40.0	*3.7 (1.0–13.2)*	11	73.3	*8.2 (2.2–30.4)*	15	100.0	–
Laboratory	28	7	25.0	1.8 (0.6–5.7)	16	57.1	*4.0 (1.5–10.6)*	25	89.3	*11.4 (3.0–42.4)*
Epidemiology	52	8	15.4	1	13	25.0	1	22	42.3	1
Specimen collection/use										
New specimen collection	58	20	34.5	2.1 (0.7–6.4)	44	75.9	*10.0 (3.3–29.8)*	52	89.7	*8.0 (2.5–25.3)*
Archived specimen (linked)	11	2	18.2	0.9 (0.1–5.5)	0	0.0	–	6	54.6	1.1 (0.3–4.6)
Archived specimen (unlinked)	20	4	20.0	1.0 (0.2–4.3)	5	25.0	1.1 (0.3–4.1)	7	35.0	0.5 (0.1–1.7)
Medical records	25	5	20.0	1	6	24.0	1	13	52.0	1

		Informed consent process			Data collection and analysis			Facility and support		
Study locations										
International										
Y	5	4	80.0	2.0 (0.2–18.3)	3	60.0	0.8 (0.1–4.8)	3	60.0	*6.7 (1.0–42.6)*
N	109	73	67.0	1	72	66.1	1	20	18.4	1
Multi-centre/-site										
Y	15	13	86.7	3.6 (0.8–16.7)	13	86.7	3.9 (0.8–18.2)	8	53.3	*6.4 (2.0–20.3)*
N	99	64	64.7	1	62	62.6	1	15	15.2	1
Required other IRB review										
Y	35	30	85.7	*4.1 (1.4–11.6)*	26	74.3	1.8 (0.7–4.3)	14	40.0	*5.2 (2.0–13.7)*
N	79	47	59.5	1	49	62.0	1	9	11.4	1
Study populations										
Involved minority populations										
Y	32	31	96.9	*24.3 (3.2–186.3)*	29	90.6	*7.5 (2.1–26.8)*	14	43.8	*6.3 (2.4–16.9)*
N	82	46	56.1	1	46	56.1	1	9	11.0	1
Involved vulnerable populations										
Y	28	23	82.1	2.7 (0.9–7.9)	19	67.9	1.1 (0.5–2.8)	9	32.1	2.4 (0.9–6.5)
N	86	54	62.8	1	56	65.1	1	14	16.3	1
Clinical study designs										
Biomedical studies	19	17	89.5	*10.7 (2.2–51.2)*	15	79.0	*3.8 (1.1–12.8)*	5	26.3	2.7 (0.7–10.3)
Drug trial	15	15	100.0	–	14	93.3	*14.0 (1.7–114.4)*	6	40.0	*5.1 (1.3–19.5)*
Laboratory	28	22	78.6	*4.6 (1.6–13.3)*	20	71.4	2.5 (0.9–6.7)	6	21.4	2.1 (0.6–7.2)
Epidemiology	52	23	44.2	1	26	50.0	1	6	11.5	1
Specimen collection/use										
New specimen collection	58	53	91.4	*9.8 (2.9–32.7)*	46	79.3	2.6 (0.9–7.1)	18	31.0	2.4 (0.7–7.9)
Archived specimen (linked)	11	4	36.4	0.5 (0.1–2.3)	7	63.6	1.2 (0.3–5.1)	1	9.1	0.5 (0.1–5.3)
Archived specimen (unlinked)	20	7	35.0	0.5 (0.1–1.7)	7	35.0	0.4 (0.1–1.2)	0	0.0	–
Medical records	25	13	52.0	1	15	60.0	1	4	16.0	1

Italics denotes statistically significant values

*CI* confidence interval, *IRB* institutional review board, *OR* odds ratio

Regarding study populations, proposals involving minority populations had higher ORs than proposals that did not involve such populations, for all ethical issues apart from study rationale and significance. In contrast, proposals involving vulnerable populations had higher ORs than proposals that did not, for study rationale/significance and study design/methodology.

When comparing the four study designs using epidemiology studies as a reference group, higher ORs were reported for biomedical/clinical research for all ethical issues except study facility and supporting documents. For drug trials, higher ORs were reported for all ethical issues except study participants and informed consent process. This was because 100 % of drug trial researchers were asked for clarifications on these two ethical issues. For laboratory studies, higher ORs were reported for three ethical issues: study design and methodology, study participants, and the informed consent process.

When comparing the four types of specimen/data use using proposals with medical records/CRFs as the reference group, higher ORs were reported for studies that collected new specimens for all ethical issues except study rationale, data collection and analysis, and research facility and supporting documents. Interestingly, when comparing proposals for studies using medical records/CRFs with those using identifiable or unidentifiable stored specimens, no statistically significant differences were reported.

## Discussion

The roles and responsibilities of an REC are to ensure protection of the safety, well-being and basic rights of potential participants and participants of a research study. The REC should review the protocol and associated documents and provide opinions on three different ethical considerations, i.e. science, ethics, and data quality [33]. The classical view of research ethics is governed by four ethical principles: respect for persons, beneficence, justice, and respect for communities [39, 40]. Ethical foundations to be considered and addressed include issues on research subjects, the informed consent process, study design concept, risk–benefit ratio, vulnerable group protection and research gatekeepers [10, 41]. In this study, the ethical issues raised in malaria research proposals fell within these common principles. Approximately half of the proposals required revision and clarification on the informed consent process and study participant protection; and, for those studies that required specimen collection, the process to obtain the specimen from the study participants. Approximately one-third required information about risk–benefit and study schedule-activities. Less than one-fifth required explanation of the research objectives and a few proposals required clarification or revision of research questions and supporting documents.

Although only approximately 12 % of the proposals in the present study needed elaboration or provision of more robust information about the research questions (study rationale and significance), debate remains whether the FTM-EC has gone beyond its designated roles. The highest percentage of ethical issues raised by the FTM-EC regarding the malaria research proposals in the study related to the specimen and data collection schedule and activities. At times, some investigators have also questioned whether the ethics committee should comment on research methodology. These scientific merit and research procedure issues remain controversial internationally. It has been reported that REC members are pressured to review a wide range of issues in research proposals, needing to provide opinions ranging from research design to patient privacy and budgeting matters [9, 33]. Some research investigators believe that these are beyond the scope of research protection [9]. Others have suggested that researchers should have already thought carefully about the nature of how the study results can be generated and how they are aligned with the aims of the research. Heterogeneity of bioethics ought to be welcomed, but those involved should engage meaningfully and explicitly with questions concerning normative justification and the methodological process and about the coherence of components of their study [42]. The OHRP also notes that REC members very often ask to what degree it is his/her responsibility to review the underlying science of the proposal [43]. It has long been argued that “if it is not good science, it is not ethical” and the US federal regulations do not clearly call for REC review of the scientific validity of the research design. However, if the underlying science is inadequate, then it follows that the study is unlikely to yield important knowledge [43]. To mitigate this controversial ethical consideration made by RECs, the ORHP guideline suggests that if the REC lacks expertise in the scientific matter of the particular proposal, the REC should recognise its limits. Although REC members are not required to be experts in scientific methodology or statistics, they should have certain basic knowledge about study design, and they should consult experts if they have concerns about the research rationale and methodology that seem to pose a significant problem [43]. At the FTM, besides some FTM-EC members who have been working in malaria research over decades, there is a pool of expertise in malaria research, ranging from molecular to field studies, and several of them have been consulted for advice for any unclear or controversial matter before issuing the letter to investigators or making a final decision on the submitted proposals.

The REC has a responsibility to consider the balance between the risks and benefits of the research proposal. International standards clearly state that the REC must safeguard the rights, safety and well-being of all study participants [10, 33, 44]. Based on the classic ethics principle of beneficence, researchers have an obligation not to harm needlessly and to promote the good of the study participants where possible. Regarding justice, researchers have an obligation to ensure that study procedures for the selection of research subjects are equitable [44]. It is also agreed that researchers neither exploit the vulnerable nor exclude unreasonably those who could be receive benefit from the study. This means that eligibility criteria listed in the proposal must be clearly justified [45]. Among all 114 malaria proposals submitted for FTM-EC review, about 28 % involved minority populations along the country borders, and 27 % involved other vulnerable groups (pregnant women, children, the elderly or unconscious patients). Thus, the finding that over one-third of proposals required clarification on the risk–benefit balance is not surprising.

As clearly stated in international standards on ethical review of research protocols [12, 23–25, 46], the informed consent process is one of the main ethical considerations that must be observed by RECs. Indeed, it could be said that the informed consent process is a legal, ethical, and regulatory requirement for most research and healthcare transactions [20, 47]. The informed consent process is based on the classic ethical principle of respect for persons, such that researchers must ensure that potential study participants make their own decisions whether or not to take part [44]. It should also be noted that, by signing informed consent documents, the study participants have agreed to a controlled breach of their privacy/confidential information for a specific purpose mentioned in the study protocol, and for use over a specific period of time [14]. It is thus important that participants are clearly informed about the methods of handling and use of their personal data, the justification for requesting or obtaining their data from different sources, and the duration of data use and storage, while maintaining their right to withdraw their consent at any time [14]. All guidelines suggest that special attention should be paid to studies involving vulnerable participants who may be unable or have limited capacity to make a decision [10, 21, 48, 49]. In a study on ethical dilemmas in malaria drug and vaccine trials [1], it was stated that, in most cases, obtaining informed consent was problematic because the studies were usually conducted with patients or surrogates with limited educational attainment levels in developing countries, and thus were not able to fully understand the study protocol. Potential participants may not understand the science underlying the study and therefore be able to make proper informed decisions. The finding in that study suggests the need for a better consent processes. As suggested elsewhere in the literature, a consent process considered valid or truly informed should have the following characteristics: (1) provision of adequate information, (2) capacity to understand that information, (3) decision making voluntarily, (4) understanding of information provided, and (5) agreement to the proposed treatment or procedure [50]. It has also been suggested that the informed consent forms are usually too long and complex for a layperson to read and comprehend, but there are various methods to simplify such forms [51]. Researchers are obliged to ensure that they plan their informed consent process with care, even the complicated aspects of research, by having the information explained simply and comprehensibly to the potential study participants. In the present study, over half the malaria research proposals and all malaria drug trial proposals submitted to FTM-EC required revision of the informed consent process to meet such requirements.

When examining different research designs, it was found that proposals on laboratory-based (usually using archived specimens) and epidemiological studies had fewer ethical issues raised by the FTM-EC than clinical research and drug trials. However, “privacy and confidentiality” showed an opposite, albeit non-statistically significant, trend. This concurs with one of the main ethical considerations in all international guidelines, that possible invasions of privacy of individuals and breaches of confidentiality may arise in biomedical and behavioural/social research [22, 24, 25, 27, 46]. As suggested in the guideline on ethics of research related to healthcare in developing countries, one of the RECs’ primary tasks is to review the ethical acceptability of research proposals with special attention to the provisions for protecting the security and confidentiality of data about patients [7]. From a data protection and privacy issues point of view, all study participants must be informed about not only what they have to do in the research, if they decide to participate, but also what and how the research plans to use the data that they provide [46]. There were reports of potential improper use or misuse of the collected data; even in case studies showing that what seems to be unlinked information can sometimes be taken for use out of context and lead to a personal data breach [46]. This could be because study procedures in clinical and drug trial proposals tended to be stated clearly by the study investigators in protecting participants’ personal information, whereas proposals of laboratory (predominantly using stored specimens) and epidemiology studies (predominantly using medical records and CRFs in healthcare settings) were generally not as clear. As suggested in the literature about ethical and legal issues of research using human specimens and clinical data, materials should be provided to the investigator with the minimal clinical information needed for the study and specimens should not be individually identifiable where applicable [15, 16, 20].

The malaria proposals that required additional review by other RECs, whether international or local, appeared to raise more ethical issues regarding study design and methodology, study participants, informed consent processes and study facility and supporting documents. This might be because such proposals are likely to be developed by non-local investigators and thus there might be certain local sensitive issues that were overseen by the proposal developers. As suggested in the literature regarding ethical principles in conducting clinical research in developing countries, investigators should consider the principle of collaboration by developing partnerships with local researchers, policy makers and the community [2]. It is recommended that the study should respect the community’s values, culture, traditions, and social practices. Even regarding scientific validity, the research team should ensure that the scientific design and methodology has recognized social value for the primary beneficiaries of the research, and is feasible within the social, political, and cultural context, or provides sustainable improvements in the local healthcare and physical infrastructure [2].

Ethical issues that should be considered, as part of field studies of malaria in developing countries, were also discussed in terms of practical problems that arise in the course of research. These include differing circumstances in developing countries, such as standards of care, incentives and reimbursement, insurance and indemnity [28]. The local REC should ensure that local context is adequately addressed and convey their knowledge of local factors that affect human subject protection [52]. The issues raised by the FTM-EC for these international studies confirm the notion that local ethical review should consider the vulnerability of study participants, particularly in remote border areas with no or poor access to healthcare services, alongside cultural beliefs and attitudes, literacy and language [25, 28]. As has also been suggested by others, the REC should provide public assurance of such protection by ensuring that investigator(s) are suitable to conduct the study, facilities are adequate, and that the methods and materials and informed consent process are appropriate [33].

Malaria research studies conducted along the Thai borders generally involved minority populations in endemic areas. These populations are considered vulnerable, with poor/limited educational attainment and cross-border/migrant status. Therefore, it is not surprising to observe that all ethical issues (except study rationale) were raised for such proposals by FTM-EC. The principle of fair selection of study population is important to minimize risks while enhancing other critical principles of collaborative partnership and social value when the research study is taking place in local communities [2]. Balancing risk–benefit considerations, informed consent processes and having respect for recruited participants and study communities, especially among vulnerable populations, remain the major concerns of all RECs [2, 25].

There were more ethical considerations in almost all aspects for drug trials and clinical studies compared with proposals for epidemiological studies. Clinical research studies usually directly involve human subjects, either with preventive, therapeutic, or non-therapeutic procedures. In general, the study procedures in such study designs put human subjects at higher risks, thus there are more ethical concerns. The primary ethical considerations of clinical studies are competent medical treatment and care, alongside an acceptable risk–benefit balance [40, 41, 44]. However, many laboratory research studies use stored specimens, with less invasive procedures, and epidemiology studies usually employ data collection through medical records, CRFs or questionnaires. Ethical issues for the latter, therefore, mainly concern confidentiality and privacy of the study participants [25]. However, it was found that studies that collect new specimens received more comments on ethical issues. There remains debate among RECs about solutions for issues around sample export, storage, and reuse [15, 16, 34]. However, it is recommended that in order to ensure adequate protection of human research subjects participating in scientific research, RECs bear the responsibility of guaranteeing that participants are provided with sufficient detail to be able to provide informed consent as well as to understand the reality of genetic research as it is practiced [53].

## Limitations of the study

The main limitation of this study is that it is based on information from only one institution, the Faculty of Tropical Medicine, Mahidol University. It may, therefore, not be representative of RECs elsewhere in Thailand or around the world where malaria proposals are submitted. However, the top five institutions publishing malaria research papers during 2010–2014 were the London School of Hygiene and Tropical Medicine (882), the University of Oxford (766), the University of Liverpool (485), Mahidol University (450, including 391 from the FTM), and Johns Hopkins University (409). The proposals under the FTM affiliation were all reviewed by the FTM-EC. This study examined all 114 malaria research proposals submitted to an REC in Thailand during a five-year period. Overall, the FTM makes a considerable contribution to malaria research globally. Identifying the ethical issues considered during the protocol review process at the FTM may inform the planning of future malaria research studies in endemic countries in Southeast Asia and/or beyond.

## Conclusions

Regardless of study design and setting, the REC focus is on the science, ethics and quality assurance of each study protocol. Several important ethical issues were identified for protocol approval, ranging from study design to supporting documents. Ethical considerations, particularly for clinical research and drug trials appeared to be mainly focused on the risk–benefit balance, vulnerable participants, and informed consent process, whereas the main considerations for laboratory and epidemiology studies were the confidentiality and privacy of data and use of specimens. As with studies subject to review by any REC, generic ethical principles are applied to malaria research, such that research validity and quality must be maintained while respecting study participants within the social, cultural and economic contexts where the research is conducted. Ethics and bioethics represent large domains of their own in balancing both theoretical and practical aspects of human research study conduct. While stakeholders, including researchers, sponsors and RECs, have to consider the practicability of research conduct within malaria-endemic settings, which are commonly populated with those who are poor and vulnerable, they cannot violate the classic ethical principles of autonomy, beneficence, and justice.

## Abbreviations

CRF: Case record form
FTM: Faculty of Tropical Medicine
FTM-EC: Faculty of Tropical Medicine Ethics Committee
FWA: Federal-wide Assurance
IRB: Institutional Review Board
NIH: National Institutes of Health
OHRP: Office for Human Research Protections
OR: Odds ratio
REC: Research Ethics Committee
